# The role of microbiota and inflammation in self-judgement and empathy: implications for understanding the brain-gut-microbiome axis in depression

**DOI:** 10.1007/s00213-019-05230-2

**Published:** 2019-04-07

**Authors:** N. Heym, B. C. Heasman, K. Hunter, S. R. Blanco, G. Y. Wang, R. Siegert, A. Cleare, G. R. Gibson, V. Kumari, A. L. Sumich

**Affiliations:** 1 Division of Psychology, Nottingham Trent University, Nottingham, NG1 4FQ UK; 2 Division of Sports Science, Nottingham Trent University, Nottingham, UK; 3 Department of Psychology, Auckland University of Technology, Auckland, New Zealand; 4 Institute of Psychiatry, Psychology and Neuroscience, King’s College London, London, UK; 5 Food and Nutritional Sciences, University of Reading, Reading, UK; 6 Centre for Cognitive Neuroscience, Brunel University London, Uxbridge, UK

**Keywords:** Brain-gut-microbiota axis, *Lactobacillus*, Inflammation, Depression, Self-judgement, Cognitive empathy, affective empathy, over-identification

## Abstract

**Rationale:**

The gut-brain axis includes bidirectional communication between intestinal microbiota and the central nervous system. *Bifidobacterium* and *Lactobacillus* spp. have been implicated in psychological health, such as depression, through various pathways (e.g. inflammation). Research needs a better understanding of direct and indirect effects through examination of psychological factors that make people susceptible to, or offer protection against, depression.

**Objective:**

This study investigated the relationships between gut microbiota, inflammation and psychological risk and resilience factors for depression.

**Methods:**

Forty participants (13 m/27 f) recruited from the general population completed self-report questionnaires for depression, self-judgement, over-identification and affective and cognitive empathy. Faecal and blood samples were taken to assay microbiota (*Bifidobacterium*; *Lactobacillus* spp.) and pro-inflammatory molecules (C-reactive protein, CRP and interleukin-6, IL-6), respectively.

**Results:**

Hierarchical regression analyses (controlling for sex, age and the shared variance of risk and resilience factors) showed that (i) cognitive depression was significantly predicted by negative self-judgement and reduced cognitive empathy; (ii) abundance of *Lactobacillus* spp. was directly related to positive self-judgement but only indirectly to cognitive depression and lower affective empathy (both through self-judgement); and (iii) CRP was the strongest predictor of reduced cognitive empathy, with suppression effects seen for age (negative) and IL-6 (positive) after controlling for CRP.

**Conclusions:**

Findings suggest that lactobacilli and inflammation may be differentially associated with mood disorder via brain mechanisms underpinning self-judgement and cognitive empathy, respectively. Further trials investigating interventions to increase *Lactobacillus* spp. in depression would benefit from direct measures of self-judgement and affective empathic distress, whilst those that aim to reduce inflammation should investigate cognitive empathy.

**Electronic supplementary material:**

The online version of this article (10.1007/s00213-019-05230-2) contains supplementary material, which is available to authorized users.

## Introduction

With a lifetime prevalence of 25%, depression is the leading cause of disability worldwide, accounting for 33% of the total disability costs in Europe (1% GDP) (Sobocki et al. 2006; WHO 2017). Fundamental issues for clinical management with pharmaceuticals include limited efficacy (approximately 30–50% of patients remain intolerant or unresponsive to pharmacotherapy), costs and side effects (Epstein et al. 2014). As such, it is imperative for global wellbeing and economy that more evidence-based and effective management techniques are developed with fewer side effects, rapid onset of therapeutic action and lower cost. Recent evidence indicates potential clinical advantage (e.g. improving mood and emotional processing) of targeting the gut-brain axis through modulation of the microbiota that inhabit the gut (Fond et al. 2015; Liu et al. 2015; Steenbergen et al. 2015; Tillisch et al. 2013).

The gut-brain axis includes bidirectional interactions between intestinal microbiota and the central nervous system (Dinan and Cryan 2017). In addition to being regulated by the brain (e.g. in response to states of stress or fear; Bailey et al. 2006), the balance between various competing microbiota species influences neural function both directly via the vagus nerve (Bravo et al. 2011) and indirectly through the immune system, neurotransmitter synthesis, endocrine function (e.g. sex and stress hormones) and other signalling molecules (e.g. Fond et al. 2015; Janik et al. 2016; Liang et al. 2015; Liu et al. 2016; Wang et al. 2016). Whilst much of our understanding about the brain-gut-microbiota axis is derived from preclinical and animal studies, which implicate an important role for *Bifidobacterium* and *Lactobacillus* genera in mood regulation, this has been broadly supported by recent human studies (Dinan and Cryan 2017; Aizawa et al. 2016; Pirbaglou et al. 2016; Wallace and Milev 2017; Rieder et al. 2017; Ng et al. 2017; Tillisch et al. 2013). For example, reduced levels of gut bifidobacteria and lactobacilli have been observed in people with major depressive disorder (Aizawa et al. 2016). Understanding the psychological mechanisms that underpin these associations would allow for the advancement of more sensitive psychological targets in clinical trials (Allen et al. 2017).

Probiotic, prebiotic and synbiotic (i.e. combined pro- and prebiotics) trials in humans to increase gut bifidobacteria and lactobacilli have met with some success in ameliorating symptoms of depression and anxiety in both non-clinical (healthy volunteers) and clinical (e.g. major depressive disorder, irritable bowel syndrome, chronic fatigue syndrome) populations (Pirbaglou et al. 2016; Rieder et al. 2017). However, negative findings also exist, including some larger trials (Romijn et al. 2017; Benton et al. 2007), possibly in part reflecting the multifarious nature of depression and anxiety, as well as the different approaches to fortify lactobacilli. Thus, whilst a synbiotic supplementation designed to increase bifidobacteria and lactobacilli did not lower depression and anxiety per se, an improvement was seen in cognitive reactivity (e.g. rumination) during sadness (Steenbergen et al. 2015)*.* In another study, a significant psychological response to supplementation with *Lactobacillus helveticus* and *Bifidobacterium longum* was only seen in a subgroup of participants with high baseline vitamin D (Romijn et al. 2017). In a trial comparing the effects of two prebiotics, namely fructooligosaccharides and galactooligosaccharides, on performance in several experimental tasks designed to index mood, a shift in emotional bias towards attending to positive words (dot-probe-test) and a reduction in salivary cortisol compared to placebo were observed (Schmidt et al. 2015). These findings suggest more complex relationships between the gut microbiota and depression—likely with specific aspects of depression or indirectly through associated psychological risk factors.

Recent models highlight the interplay between empathy and self-compassion in the development of mood disorders, including depression (MacBeth and Gumley 2012; Schreiter et al. 2013). Affective empathy refers to the tendency to feel another person’s emotions, sometimes referred to as emotional contagion or responsivity (Reniers et al. 2011). Capacity for affective empathy protects against some maladaptive personality traits (e.g. psychopathy, narcissism) and promotes prosocial behaviour, but also carries risk. Taking on empathic distress from others through heightened affective empathy in combination with over-identification—that is being self-absorbed in one’s emotional reaction and suffering (as opposed to self-compassion)—can increase risk for depression, emotional burnout and compassion fatigue. Schreiter et al. (2013) propose that greater capacity for affective empathy might underpin greater risk of depression in women. On the other hand, the ability to conceptualise the distinct perspective of another person (cognitive empathy) and certain dimensions of self-compassion, such as positive self-judgement, protect against this risk (Schreiter et al. 2013). Indeed, the aforementioned implication of lactobacilli in promoting positive emotional bias (Schmidt et al. 2015) and reducing rumination (Steenbergen et al. 2015) may well be reflected in a positive view towards oneself (i.e. positive self-judgement). To our knowledge, the relationships between these psychological risk and resilience factors for mood disorder and the microbiota have not been directly investigated.

One of the mechanisms through which the microbiota might interact with mood is via the immune system (Rea et al. 2016). For example, bifidobacteria and lactobacilli have anti-inflammatory effects, which may underpin their anti-anxiety and anti-depressant action (Maldonado Galdeano et al. 2015). The relationship between the immune response and depression has also been proposed to be bidirectional. Thus, chronic stress induces elevated hormone (e.g. adrenaline, noradrenaline and cortisol) release, via the persistent activation of the hypothalamic-pituitary-adrenal axis and autonomic nervous system that affects the neural regulation of emotions and immune system function (Kiecolt-Glaser et al. 2015). On the other hand, chronic activation of inflammatory pathways alters synthesis of certain neurotransmitters (e.g. serotonin, dopamine), increasing stress responsivity, vulnerability to mood disorders and impaired cognitive function (Kiecolt-Glaser et al. 2015).

In light of the above findings, the current study investigated, for the first time to our knowledge, the relationship between microbiota (bifidobacteria and lactobacilli), inflammation (CRP, IL-6) and psychological risk and resilience factors for depression (self-judgement, over-identification, affective and cognitive empathy). We study these associations in the general population to investigate both psychological risk and resilience factors (e.g. positive self-judgement is a resilience factor that might be compromised in MDD), free from medication. It was hypothesised that bifidobacteria and lactobacilli would be associated with reduced depression, inflammation, over-identification and affective empathy, but increased positive self-judgement and cognitive empathy. If these associations are due to interactions with the immune system, they might be expected to be mediated by measures of inflammation.

## Materials and methods

### Participants

A total of 40 participants took part in this study—13 men (aged 22–67, mean = 36.77, SD = 13.24) and 27 women (aged 19–56, mean = 36.19, SD = 10.72; of which 2 were breastfeeding). Participants were eligible if they were aged over 18 years, had fluent English language skills and were non-smokers and predominantly right-handed. Exclusion criteria included intake of antibiotics (within the previous 3 months); regular intake of psychotropic or anti-inflammatory drugs, prebiotic/probiotic or synbiotic supplements (within the previous 4 weeks); use of laxatives, anti-diarrhoeal medications, enemas or suppositories (within the previous 4 weeks) or history of colonic irrigation (within the previous 6 months), gastrointestinal infection (within the previous 4 weeks), major surgery (within the previous 3 months), major physical illnesses including gastrointestinal, inflammatory or autoimmune diseases; and personal history of diagnosed psychiatric or neurological disorder other than depression. The majority of participants were Caucasian (*n* = 33) and had attained university-level qualification (*n* = 30; *n* = 7 A level equivalent; *n* = 3 other higher education), and were in full-time employment (*n* = 25; *n* = 12 were in full-time education). Most regularly consumed alcohol (*n* = 34), and some took regular prescribed medication (*n* = 14; *n* = 7 contraceptives, *n* = 3 blood pressure and statins; *n* = 1 thyroid; *n* = 1 stomach acid; *n* = 1 acne; *n* = 1 asthma), consumed dietary supplements (*n* = 13) and reported food allergies (*n* = 6). Two participants reported chronic depression, but were not currently taking anti-depressants. One participant reported chronic back pain and one discoid eczema, and one minor surgery (removal of nasal polyps and septoplasty) within the previous 6 months.

The study procedures were approved by the Business, Law and Social Sciences ethics committee, Nottingham Trent University. Written informed consent was provided by all participants.

### Psychometrics

#### Depression

The Beck Depression Inventory II (BDI-II) has 21 items assessing cognitive (8 items, *α* = 0.81) and non-cognitive (13 items, *α* = 0.87) domains, with statements ranging in intensity from 0 to 3. Participants were asked to endorse the most characteristic statements over the past 2 weeks. Domain subscores were calculated with higher scores denoting more severe depressive symptoms. The BDI-II has been validated for clinical and non-clinical adolescents and adults (Beck et al. 1996; Steer et al. 1999). The BDI-II total and factor scores show good reliability (Cronbach’s alphas, *α*, ranging between 0.74 and 0.93) and construct validity (Steer et al. 1999).^1^

#### Self-judgement and over-identification

Subscales of the self-compassion scale (Neff 2003) were used to measure self-judgement (5 items, e.g. “I’m disapproving and judgmental about my own flaws and inadequacies”, *α* = 0.77) and over-identification (4 items, e.g. “When something upsets me I get carried away with my feelings”, *α* = 0.81). Items are rated using a Likert scale ranging from 1 (almost never) to 5 (almost always). Scores were reverse coded such that higher scores reflect greater self-compassion (more positive self-judgement; less over-identification, respectively). The scales have shown good internal and test-retest (*r*s = 0.88) reliability and discriminant validity (Neff 2003).

#### Empathy

The questionnaire for cognitive and affective empathy (Reniers et al. 2011) is a 31-item scale, containing cognitive empathy (19 items) and affective empathy (12 items). Items are rated using a Likert scale from 1 (strongly agree) to 4 (strongly disagree) and computed such that higher scores indicate greater empathy. Previous Cronbach’s alphas for subscales ranged from 0.65 to 0.85 (Reniers et al. 2011).

### Procedure

Participants in the current study form a subgroup of a larger ongoing psychometric project (*N* = 400). After completion of the online screening survey, participants received further study information for the experimental parts and were invited to register interest for these. All eligible participants who did not meet any exclusion criteria and resided near the laboratory location were invited to participate in this study. For the current study part, participants were required to provide blood and stool samples. Psychometric and laboratory testing interval ranged from 0 to 78 days apart (mean = 35.12 days; SD = 22.69).

#### Faecal samples

Participants were issued with a Fecotainer® (www.fecotainer.eu/) and detailed instructions regarding stool collection. Twenty-eight out of 40 participants were able to provide a sample on the day of testing; the remaining 12 took the Fecotainer® home and provided it at the earliest possible opportunity. Stool samples were aliquoted (1 g per participant) and frozen at − 20 °C within 2 h of voiding and transported to the lab for further processing.

#### Blood samples

Ten milliliters of blood was drawn from the antecubital fossa using BD Vacutainer® Safety Lok™ blood collection sets containing ethylenediaminetetraacetic acid (EDTA) as an anticoagulant, and immediately placed on ice. Blood samples were centrifuged for 15 min (1000×*g* at 4 °C) within 30 min of collection. The plasma fraction was then aliquoted into cryotubes and stored at − 80 °C until analysis.

### Processing of biosamples

#### Faecal sample preparation

Stool samples were thawed and re-suspended in phosphate-buffered saline (PBS; 0.01 M phosphate buffer, 0.0027 M potassium chloride and 0.137 M sodium chloride) and homogenised in a stomacher for 2 min at 460 paddle beats per minute. The resulting faecal slurry was vortexed with 3-mm glass beads (VWR) for 30 s before being centrifuged at 400×*g* for 2 min at room temperature. The supernatant (375 μL) was fixed in 4% (*w*:*v*) (1125 μL) paraformaldehyde for 4 h at 4 °C. To wash the cells out of paraformaldehyde, samples were centrifuged at 13,000×*g* in 1 mL PBS for 5 min at room temperature; this centrifugation was repeated two more times, then samples were re-suspended in 150 μL PBS and stored in ethanol (1:1 by *v*:*v*) at − 20 °C for enumeration by fluorescence in situ hybridisation (FISH).^2^

#### Flow cytometry-fluorescence in situ hybridisation for enumeration of faecal microbial population

Flow-FISH, using fluorescently labelled 16S rRNA–targeted oligonucleotide probes (Sigma-Aldrich, Steinheim, Germany) labelled at the 5′ end with fluorochrome Alexa®647, was used for bacterial enumeration of the faecal samples. A panel of 11 commonly used 16S rRNA oligonucleotide probes targeting specific groups of bacteria was used. However, only Bif164 (bifidobacteria) and Lab158 (lactobacilli) were investigated in the current study. The EUBmix probe (linked to the fluorochrome Alexa®488) was used to measure total bacteria within the samples via a fluorescence detector. From this, bacterial groups of interest were assessed using probes (linked to fluorochrome Alexa®647) to fluoresce at a different excitation wavelengths to be detectable in a fluorescence detector (FL4-H).

Fixed samples were treated with lysozyme TE-FISH buffer (0.1 M Tris, 0.05 M EDTA, 1 mg/mL lysozyme) for 10 min at room temperature. The sample was washed twice in PBS and re-suspended in hybridization buffer (0.9 M NaCl, 0.02 M Tris/HCl, 30% formamide, 0.01% SDS), together with the oligonucleotide probe of interest. The hybridising mixtures were incubated overnight at 35 °C before 150 μL of hybridisation buffer was added to each tube, mixed and centrifuged 3 min at 13,000×*g*. Supernatant (150 μL) was taken off and discarded using a pipette. The sample was again centrifuged for 3 min 13,000×*g* and all the supernatant removed. The pellet was re-suspended in PBS solution and the sample was read using the accuri C6 (BD Biosciences, Oxford, UK) flow cytometer.

#### ELISA analysis

Plasma samples and reagents were brought to room temperature prior to analysis. Plasma was diluted 100-fold (CRP only) and assayed using a 96-well solid-phase quantitative sandwich enzyme immunoassay (R&D Systems Quantikine® ELISA). The intra- and inter-assay precision was less than 10% for both CRP and IL-6, and the mean minimum detectable level of the assay was 0.010 ng/mL for CRP and 0.039 pg/mL for IL-6 (all samples exceeded those apart from one IL-6 sample that was zero). A set of standards was prepared using calibrator diluent to make a two-fold dilution series. Fifty microliters of standard or sample was added to each well, and all standards and samples were assayed in duplicate. Duplicate readings of each standard and sample were averaged, and the average zero standard optical density was subtracted. A standard curve was generated by reducing the data using computer software (www.elisaanalysis.com) to generate a four-parameter logistic (4-PL) curve fit. Concentrations read from the standard curve were multiplied by the dilution factor in the case of CRP. Optical density was determined within 30 min using a microplate reader set to 450 nm with wavelength correction of 570 nm (CRP) and 650 nm (IL-6).

### Statistics

Pearson’s zero-order correlations were used to test the relationship between the psychometrics (cognitive depression, non-cognitive depression, self-judgement, over-identification, affective empathy, cognitive empathy) and their associations with the biomarkers (lactobacilli, bifidobacteria, CRP, IL-6). Hochberg’s correction was used to adjust for multiple comparisons. For the psychometrics, hierarchical linear regressions were conducted to assess the unique impact of the psychological risk factors on depression (controlling for age and sex). Based on the results, hierarchical linear regression was used to test for direct associations of identified biomarkers with (a) depression and (b) the identified psychological risk factors (controlling for age and sex, and related psychometric measures). There is much debate over rules-of-thumb for suitable sample size in multiple linear regression (Green 1991), which range from *n* > 5/predictor variable to *n* > 400, and as with the current study, is determined in part by pragmatics. Nevertheless, according to Green (1991), *N* = 38 is sufficient to detect large effect sizes with 1–5 predictor variables and *N* = 41 for 6 predictors. Due to small sample size, adjusted *R*^2^ values are provided in the regression analyses. Sobel’s test was used to test for mediation and/or suppression effects.

## Results

### Zero-order correlations and regression for psychometric data

Table 1 shows means, standard deviations and zero-order correlations for psychometric measures (including alphas), microbiota and inflammation. CRP^3^ (*z* = 10.32; *z*_log_ = 1.74) and cognitive depression (*z* = 5.46; *z*_log_ = 0.09) were positively skewed and were normalised through log transformation. All Cronbach’s alphas were acceptable.

**Table 1:** Means and standard deviations for microbiota, inflammation and psychometrics

	*α*	Mean	SD	cogD	ncogD	Self-j	Over-i	cogE	affE
Depression									
Cognitive^log^	0.75	2.15	2.34	1					
Non-cognitive	0.79	5.80	4.14	0.625**	1				
Self-compassion									
Self-judgement	0.73	3.02	0.68	− 0.493**	− 0.089	1			
Over-identification	0.86	2.99	0.98	− 0.372*	− 0.099	0.650**	1		
Empathy									
Cognitive	0.93	57.35	8.81	− 0.213	− 0.206	− 0.191	− 0.080	1	
Affective	0.85	32.63	5.86	0.216	0.048	− 0.499**	− 0.534**	0.235	1
Microbiota									
Bifidobacteria	–	7.79	0.59	− 0.069	0.058	0.086	0.145	0.029	− 0.160
Lactobacilli	–	7.51	0.38	− 0.333*	− 0.118	0.593**	0.357*	−0.091	− 0.444**
Inflammation									
CRP^log^	–	16.20	28.74	0.173	0.152	− 0.046	0.096	0.514**	− 0.263
IL-6	–	1.02	0.43	0.179	0.118	− 0.052	0.164	0.022	− 0.113

*N* = 40; *p < 0.05; **p < 0.01 (in italics, those that survived Hochberg’s corrections); inflammation markers in ng/mL, microbiota numbers are expressed as mean log10 cells/g fresh faeces. *cogD* cognitive depression, *ncogD* non-cognitive depression, *self-j* self-judgement, *over-i* over-identification, *cogE* cognitive empathy, *affE* affective empathy

Cognitive depression was significantly negatively correlated with positive self-judgement (*p*_adj_ = 0.006) and lower over-identification; however, the latter did not survive correction (*p*_adj_ = 0.08). Its associations with increased affective and reduced cognitive empathy did not reach significance (*p*s > 0.05). Non-cognitive depression was not associated with any of the measures. Affective empathy was significantly negatively correlated with lower over-identification (*p*_adj_ < 0.001) and positive self-judgement (*p*_adj_ = 0.007). (Scatterplots of all significant associations can be found in the supplementary material.)

#### Psychological risk factors of cognitive depression^4^

A hierarchical linear regression was conducted to test the unique contribution of psychometric risk factors predicting cognitive depression, controlling for sex and age (Table 2 for coefficient statistics) and examine any potential suppressor effects. Sex and age were entered first (model 1), followed by the self-compassion variables (model 2), and then affective and cognitive empathy (model 3). Model 1 was non-significant, explaining only 6% of the variance in cognitive depression (*R*^2^_adj_ = 0.01, SE = 0.71; sum of squares regression/residual = 1.19/18.45, df = 2/37, mean square regression/residual = 0.60/0.50, *F* = 1.20, *p* = 0.313). Model 2 was significant and explained a further 21% (*R*^2^_adj_ = 0.18.3, SE = 0.64; sum of squares regression/residual = 5.24/14.40, df = 4/35, mean square regression/residual = 1.31/0.41, *F* = 3.18, *p* < 0.05, ∆*R*^2^ = 0.20.6, ∆*F* = 4.92, *p* = 0.013). Model 3 explained only a further 0.9% of the variance (*R*^2^_adj_ = 0.24, SE = 0.62; sum of squares regression/residual = 7.06/12.58, df = 6/33, mean square regression/residual = 1.18/0.38, *F* = 3.08, *p* < 0.05, ∆*R*^2^ = 0.09, ∆*F* = 2.38, *p* = 0.108). Significant associations were only seen for positive self-judgement and cognitive empathy (both negative).

**Table 2:** Linear regression for psychometric risk factors in cognitive depression

	*B*	SE	Std. beta	*t*	*p*	Tolerance	VIF
Model 1. Constant	1.220	0.413		2.954	0.005		
Sex	0.208	0.238	0.139	0.872	0.389	0.999	1.001
Age	− 0.012	0.010	− 0.201	− 1.259	0.216	0.999	1.001
Model 2. Constant	2.913	0.658		4.425	< 0.001		
Sex	− 0.181	0.250	− 0.121	− 0.724	0.474	0.753	1.329
Age	− 0.005	0.009	− 0.084	− 0.559	0.579	0.937	1.068
Self-judgement	− 0.487	0.216	− 0.464	− 2.254	0.031	0.495	2.021
Over-identification	− 0.075	0.139	− 0.104	− 0.543	0.591	0.571	1.751
Model 3. Constant	4.238	1.214		3.490	< 0.001		
Sex	− 0.080	0.245	− 0.054	− 0.328	0.745	0.724	1.381
Age	− 0.008	0.009	− 0.124	− 0.824	0.416	0.862	1.160
Self-judgement	− 0.519	0.215	− 0.494	− 2.415	0.021	0.465	2.152
Over-identification	− 0.040	0.143	− 0.054	− 0.277	0.784	0.500	1.998
Cognitive empathy	− 0.026	0.012	− 0.323	− 2.172	0.037	0.879	1.138
Affective empathy	0.005	0.022	0.045	0.249	0.805	0.601	1.665

*N* = 40; dependent variable was cognitive depression; sex coded: 0 = male/1 = female

### Zero-order correlations and regressions for biomarkers

Abundance of *Lactobacillus* spp. was associated with increased *Bifidobacterium* spp. (*r* = 0.34, *p* = 0.034, *p*_adj_ > 0.05 n.s.) and CRP_log_ with increased levels of IL-6 (*r* = 0.37, *p* = 0.018, *p*_adj_ > 0.05 n.s.). As shown in Table 1, abundance of *Lactobacillus* spp. was associated with increased positive self-judgement (*p*_adj_ < 0.001) and lower over-identification (though this did not meet adjusted significance *p*_adj_ > 0.05 n.s.), as well as reduced affective empathy (*p*_adj_ = 0.02) and cognitive depression [log] (though this again did not meet adjusted significance *p*_adj_ > 0.05 n.s.). CRP_log_ was associated with reduced cognitive empathy (*p*_adj_ = 0.005). *Bifidobacterium* spp. and IL-6 were not associated with psychometric measures.

#### Lactobacilli in depression and self-judgement

Hierarchical linear regressions were conducted to test the independence of the relationships between psychometrics and lactobacilli, controlling for sex and age. The first regression examined the direct effect of lactobacilli on cognitive depression (Table 3). Sex and age were entered into an initial model, followed by lactobacilli (model 2) and then self-judgement and cognitive empathy (model 3). Model 1 was non-significant, explaining only 6% of the variance (*R*^2^_adj_ = 0.01, SE = 0.71; sum of squares regression/residual = 1.19/18.45, df = 2/37, mean square regression/residual = 0.59/0.49, *F* = 1.20, *p* = 0.313). Model 2 was significant and explained a further 9.8% (*R*^2^_adj_ = 0.09, SE = 0.68; sum of squares regression/residual = 3.13/16.51, df = 3/36, mean square regression/residual = 1.04/0.46, *F* = 2.27, *p* < 0.09, ∆*R*^2^ = 0.09, ∆*F* = 4.21, *p* = 0.047). Model 3 was also significant and explained a further 20% (*R*^2^_adj_ = 0.26, SE = 0.61; sum of squares regression/residual = 7.05/12.60, df = 5/34, mean square regression/residual = 1.41/0.37, *F* = 3.80, *p* < 0.008, ∆*R*^2^ = 0.20, ∆*F* = 5.29, *p* = 0.01). The significant associations for lactobacilli in model 2 (*p* < 0.05) became non-significant (*p* = 0.67) when positive self-judgement and cognitive empathy were added to the model 3 (both significantly negatively related to cognitive depression).

**Table 3:** Linear regression showing indirect relationship between lactobacilli and cognitive depression

	*B*	SE	Std. beta	*t*	*p*	Tolerance	VIF
Model 1. Constant	1.220	0.413		2.954	0.005		
Sex	0.208	0.238	0.139	0.872	0.389	0.999	1.001
Age	− 0.012	0.010	− 0.201	− 1.259	0.216	0.999	1.001
Model 2. Constant	5.786	2.260		2.560	0.015		
Sex	0.115	0.233	0.077	0.494	0.624	0.962	1.040
Age	− 0.013	0.009	− 0.205	− 1.339	0.189	0.999	1.001
Lactobacilli	− 0.598	0.292	− 0.320	− 2.052	0.047	0.962	1.039
Model 3. Constant	5.351	2.325		2.301	0.028		
Sex	− 0.051	0.242	− 0.034	− 0.212	0.833	0.718	1.392
Age	− 0.008	0.009	− 0.128	− 0.880	0.385	0.891	1.122
Lactobacilli	− 0.142	0.329	− 0.076	− 0.433	0.668	0.612	1.634
Self-judgement	− 0.521	0.213	− 0.496	− 2.442	0.020	0.458	2.184
Cognitive empathy	− 0.026	0.012	− 0.320	− 2.245	0.031	0.926	1.080

*N* = 40; dependent variable was cognitive depression (log); sex coded: 0 = male/1 = female

Following this, a hierarchical regression was run to examine the direct effects of lactobacilli on positive self-judgement (controlling for sex and age, and related psychometric measures; Table 4)*.*^5^ Sex and age were entered into an initial model predicting positive self-judgement, followed by cognitive depression, low over-identification and affective empathy (model 2)^6^ and finally lactobacilli (model 3). Model 1 was significant and explained 28% of the variance in self-judgement (*R*^2^_adj_ = 0.24, SE = 0.59; sum of squares regression/residual = 4.96/12.83, df = 2/37, mean square regression/residual = 2.48/0.35, *F* = 7.15, *p* = 0.002). Model 2 explained a further 31% (*R*^2^_adj_ = 0.53, SE = 0.46; sum of squares regression/residual = 10.54/7.25, df = 5/34, mean square regression/residual = 2.11/0.21, *F* = 9.89, *p* < 0.001, ∆*R*^2^ = 0.31, ∆*F* = 8.73, *p* < 0.001). Inclusion of lactobacilli (model 3) explained a further 8.7% (*R*^2^_adj_ = 0.62, SE = 0.42; sum of squares regression/residual = 12.10/2.01, df = 6/33, mean square regression/residual = 2.01/0.17, *F* = 11.69, *p* < 0.001, ∆*R*^2^ = 0.09, ∆*F* = 9.01, *p* = 0.005). Significant associations were seen for sex (higher in men), cognitive depression (negative in model 2, but becomes n.s. in model 3), low over-identification (positive in models 2 and 3) and lactobacilli (positive in model 3).

**Table 4:** Linear regression showing direct relationship between lactobacilli and positive self-judgement

	*B*	SE	Std. beta	*t*	*p*	Tolerance	VIF
Model 1. Constant	3.000	0.34		8.71	< 0.001		
Sex	− 0.680	0.20	− 0.480	− 3.40	0.002	1.00	1.00
Age	0.010	0.01	0.220	1.57	0.13	1.00	1.00
Model 2. Constant	3.232	0.712		4.536	0.000		
Sex	− 0.353	0.171	− 0.248	− 2.066	0.047	0.831	1.204
Age	0.008	0.007	0.134	1.171	0.250	0.910	1.099
Cog-depression_log_	− 0.251	0.114	− 0.264	− 2.205	0.034	0.837	1.195
Over-identification	0.230	0.099	0.333	2.331	0.026	0.588	1.702
Affective empathy	− 0.022	0.015	− 0.193	− 1.436	0.160	0.666	1.502
Model 3. Constant	− 1.847	1.810		− 1.021	0.315		
Sex	− 0.346	0.154	− 0.243	− 2.251	0.031	0.831	1.204
Age	0.009	0.006	0.149	1.441	0.159	0.908	1.102
Cog-depression_log_	− 0.175	0.106	− 0.184	− 1.657	0.107	0.789	1.268
Over-identification	0.208	0.089	0.301	2.337	0.026	0.584	1.714
Affective empathy	− 0.009	0.015	− 0.077	− 0.608	0.547	0.604	1.655
Lactobacilli	0.613	0.204	0.344	3.001	0.005	0.738	1.356

*N* = 40; dependent variable was positive self-judgement; sex coded: 0 = male/1 = female

#### Inflammation in cognitive empathy

A hierarchical regression was run to examine the direct effects of inflammation on cognitive empathy (controlling for sex and age; Table 5)*.*^7^ In model 1, sex and age alone were not significantly associated with cognitive empathy. Model 2, including CRP_log_ and IL-6, significantly explained 44% of the variance in cognitive empathy (*R*^2^_adj_ = 0.37, SE = 0.6.97; sum of squares regression/residual = 1326.57/1698.53, df = 4/35, mean square regression/residual = 331.64/48.53, *F* = 6.83, *p* = 0.001, ∆*R*^2^ = 0.37, ∆*F* = 11.46, *p* < 0.001). CRP_log_ remained the strongest predictor of cognitive empathy. In addition, age showed a negative and IL-6 a trend for a positive association with cognitive empathy, suggesting suppression effects, which were confirmed using Sobel’s tests for age (*p* = 0.039) and IL-6 (*p* = 0.028).

**Table 5:** Linear regression showing direct relationship between C-reactive protein (CRP) and cognitive empathy

	*B*	SE	Std. beta	*t*	*p*	Tolerance	VIF
Model 1. Constant	57.39	5.10		11.25	< 0.001		
Sex	4.46	2.94	0.24	1.51	0.14	1.00	1.00
Age	− 0.08	0.12	− 0.11	− 0.69	0.50	1.00	1.00
Model 2. Constant	66.89	5.87		11.39	< 0.001		
Sex	3.38	2.40	0.18	1.41	0.17	0.96	1.04
Age	− 0.23	0.10	− 0.30	− 2.25	0.03	0.88	1.14
CRP_log_	− 4.67	0.98	− 0.69	− 4.77	< 0.001	0.77	1.30
IL-6	5.35	2.83	0.26	1.89	0.07	0.85	1.18

*N* = 40; dependent variable was cognitive empathy; sex coded: 0 = male/1 = female

## Discussion

The current study investigated the relationship between microbiota, inflammation and psychological risk factors for depression, including poor self-judgement, over-identification, high affective empathy and low cognitive empathy, in a non-clinical sample. Multiple linear regression supported a significant role for negative self-judgement and poor cognitive empathy in explaining variance in cognitive depression. As expected, the microbiota markers (bifidobacteria and lactobacilli) were positively correlated with each other and the same was seen for the inflammation markers (CRP and IL-6). Although these associations did not survive Hochberg’s correction, it is likely these reflect true relationships of small-to-modest effect size and not statistical errors. Whilst *Bifidobacterium* spp. and IL-6 were not associated with psychometric measures, the other two markers (*Lactobacillus* spp., CRP) showed some distinct associations. Faecal lactobacilli concentrations were strongly correlated with positive self-judgement and lower affective empathy, and marginal correlations were seen with lower over-identification and cognitive depression, though these did not survive correction for multiple comparisons. A follow-up hierarchical regression confirmed that the significant association of lactobacilli with cognitive depression (after controlling for age and sex) became non-significant once self-judgement was taken into account. Despite a relatively small sample size with limited psychometric and biometric range of scores, linear regression confirmed a strong direct relationship between positive self-judgement and *Lactobacillus* spp. after controlling for sex, age, cognitive depression, over-identification and affective empathy. CRP was inversely associated with cognitive empathy, but not related with either bifidobacteria or lactobacilli abundance.

To our knowledge, this is the first study to show a relationship of *Lactobacillus* spp. with positive self-judgement and reduced affective empathy. Results are in line with previous studies implicating the brain-gut-microbiota axis in cognitive reactivity to sadness (e.g. rumination) (Steenbergen et al. 2015) and emotional bias (Schmidt et al. 2015). Future work will need to investigate whether shared variance of affective empathy and self-judgement with lactobacilli reflects shared neural mechanisms*.* For example, depression, affective empathy and poor self-compassion are linked to hypersensitivity of the amygdala and amygdala-hippocampal connectivity (Doerig et al. 2016). Furthermore, activation of the medial prefrontal cortex is implicated in self-criticism (i.e. poor self-judgement) during error processing (Longe et al. 2010), affective empathy (resting-state fMRI; Luo et al. 2018) and depression (Murray et al. 2011). Therefore, either or both of these mechanisms may mediate the relationships between affective empathy, self-judgement and lactobacilli.

Growing literature supports an effect of microbiota on brain networks involved in response to and regulation of negative affect (e.g. response to threat, fear, sadness). For example, in animals, *Lactobacillus* spp. administration and/or promotion ameliorates adverse effects of stress on hippocampal serotonin, norepinephrine and brain-derived neurotrophic factor (Liang et al. 2015) and increases hippocampal and prefrontal *N*-acetyl-aspartate, GABA and glutamate: biomarkers that are associated with depression (Janik et al. 2016). Also, supplementation with *Bifidobacterium longum* improved hippocampal-dependent memory and altered frontal midline EEG, increasing electroencephalographic mobility (Allen et al. 2016). An effect of the microbiota on amygdala sensitivity to negative emotions (e.g. fear) has been observed in rats (Hoban et al. 2017) and in humans with irritable bowel syndrome (but with no concurrent effect on inflammatory markers (Pinto-Sanchez et al. 2017)). Furthermore, reduction in activity in various emotion and viscerosensory networks (e.g. amygdala, insular and periaqueductal grey) during an attentional emotion processing task was seen in healthy women, following 4-week consumption of fermented milk containing *Lactobacillus bulgaricus*, *Bifidobacterium lactis* and *Streptococcus thermophilus* (Tillisch et al. 2013). Moreover, the HPA cortisol release response is attenuated by a prebiotic that increases *Lactobacillus* (Schmidt et al. 2015). Another neuroimaging study found differences in brain structure and function—in regions associated with emotional, attentional, and sensory processing—depending on whether women had higher concentrations of *Bacteroides* spp. or *Prevotella* spp. (Tillisch et al. 2017). Further neuroimaging studies are needed to investigate these limbic networks in relation to lactobacilli and psychological mechanisms underpinning depression in humans, such as self-judgement, over-identification and cognitive and affective empathy. Such studies should also investigate the role of *Lactobacillus* and the peripheral nervous system in subjective aspects of visceral interoception and enhanced empathy (Critchley and Harrison 2013).

Alternatively, the association between lactobacilli and self-judgement may reflect an effect of psychological state on the microbiota. That is, in animal models, stressor exposure has been shown to disrupt the microbial population associated with the colonic mucosa, reducing *Lactobacillus* abundance (Galley et al. 2014). If this were the case, a stronger association might have been expected between general depression and lactobacilli. Nevertheless, the current study is limited in determining the direction of the identified relationships, and therefore, further longitudinal studies are needed. In the current study, the largest effects were seen for a more specific facet of depression (cognitive depression) and associated personality traits, self-judgement, affective empathy and over-identification. Future studies, including trials, should thus use scales that load more on these aspects of depression. Trials that either manipulate the microbiota to affect self-judgement and empathy or manipulate these psychological constructs to affect the microbiota are needed to better understand direction of the currently observed relationships.

Self-judgement or affective empathy was not associated with inflammatory cytokines. Thus, current data cannot support the idea of an inflammatory mechanism underpinning the link between microbiota and these psychological constructs; however, we only investigated two molecules. A recent meta-analysis suggests differentiation between inflammatory markers and subtypes of depression (melancholic, non-melancholic; Yang et al. 2018). Here, IL-6 and IL-1β were increased in melancholic subtypes, whilst CRP was higher in non-melancholic forms. Future studies may investigate other markers of immune response: both pro-inflammatory and anti-inflammatory in relation to dispositional traits. Furthermore, very small doses of microbes within the gastrointestinal tract affect neurotransmission in several brain regions (i.e. paraventricular hypothalamus, central nucleus of the amygdala, bed nucleus of the stria terminalis), independent to the activation of an immune response (Goehler et al. 2007). Thus, alternative pathways might be through a more direct role in the synthesis of neurotransmitters and their precursors and/or via the vagus nerve (Bravo et al. 2011) and require further investigation.

CRP did show a strong negative association with cognitive empathy, independent of age and sex. This is in line with studies showing high levels of CRP in autism, which has been associated with poor cognitive empathy (Khakzad et al. 2012). Also, inflammatory challenge by endotoxin impaired performance on an experimental measure of cognitive empathy (i.e. theory-of-mind) (Moieni et al. 2015). Together with our findings, this suggests that even in the general population, the presence of inflammation can impair understanding of other people’s perspectives. Autism, in some cases or in some part, has been proposed to be underpinned by microbial dysbiosis (Liu et al. 2015). However, the current data do not support the idea that this is through an association between *Lactobacillus* spp. and cognitive empathy, and future studies should investigate microbiota in relation to other dimensions of the autistic spectrum (e.g. weak central coherence, hyper-systematised thinking, poor executive control). Currently, it is proposed that inflammation and lactobacilli may be differentially associated with mood disorder via networks underpinning cognitive empathy and self-judgement, respectively.

Furthermore, behavioural effects of members of *Bifidobacterium* and *Lactobacillus* can depend on the specific species or strain. For example, Bifidobacteria can have strain-specific intrinsic effects on different types of stress-induced behaviours and physiological responses in BALB/c mice (Savignac et al. 2014). Thus, although bifidobacteria were unrelated to psychometric measures in the current study, future work should investigate specific strains of *Bifidobacterium* (and those of *Lactobacillus*) and their association with psychometric risk for depression.

After controlling for CRP levels, the non-significant zero-order association between IL-6 and cognitive empathy moved towards a positive association, suggesting a suppression effect, and this shift was confirmed through a significant Sobel test. Similarly, age showed a negative association with cognitive empathy once CRP levels were controlled. Reduction in cognitive, but not affective empathy, in late adulthood has been previously documented (Bailey et al. 2008). Regarding IL-6, high levels are usually associated with depression and poor cognition, at least in clinical and ageing populations (see Wang et al. 2017 for review). However, some evidence suggests a positive association between IL-6 and cognitive function in some populations, and an inverted U function has been proposed, such that cognition is improved at moderate IL-6 levels but adversely affected at very low or very high levels (Kozora et al. 2001; Wang et al. 2017). Thus, the positive association (after controlling for CRP) between IL-6 and cognitive empathy warrants further investigation in the general population compared to clinical groups.

In summary, strong relationships exist between *Lactobacillus* spp. and self-judgement, and between CRP and cognitive empathy. Larger studies are needed, ideally including clinical samples (Ng et al. 2017), to confirm associations between other variables that did not survive correction for multiple comparisons. These findings suggest further trials investigating interventions to increase lactobacilli in depression would benefit from direct measures of self-judgement and affective empathic distress, whilst those that aim to reduce inflammation should investigate cognitive empathy. Finally, future trials should also investigate whether increasing gut bifidobacteria and lactobacilli boosts clinical effects of psychological therapies (e.g. self-compassion-focused therapies), through decreased rumination and positive (or less negative) self-judgement, especially in people with drug-resistant depression.

## Electronic supplementary material

ESM 1 (DOCX 129 kb)

## Footnotes

1 28 participants (70%) scored in the normal range (between 0 and 10), 11 (17.5%) showed mild mood disturbance (11–16) and 1 (2.5%) participant showed moderate depression (score of 30).

2 Sequencing has been shown to underestimate bifidobacteria (Sim et al. 2012; Walker et al. 2015), which were key to our interests. FISH is an established technique which provides absolute quantitative measures (rather than ratios or abundance) and has been the preferred method for several recent studies (e.g., Costabile et al. 2016).

3 There was one outlier for CRP (164.85 ng/mL); however, its removal did not meaningfully change the associations with the psychometric measures.

4 Regression for non-cognitive depression is not reported as all zero-order correlations and the regression models were non-significant.

5 Following the same rationale, similar hierarchical linear regressions were conducted to test whether lactobacilli have a direct relationship to (a) low over-identification and (b) affective empathy, respectively; however, no significant associations were seen for lactobacilli after controlling for sex, age and related psychometric measures. Significant associations were seen only for (a) positive self-judgement (positive) and affective empathy (negative) predicting lower over-identification, and (b) lower over-identification (negative) predicting reduced affective empathy.

6 These were entered as predictors as they were significantly correlated with both positive self-judgement and lactobacilli.

7 As there were no significant associations with CRP or cognitive empathy, no other psychometric measures were added; however, IL-6 was entered as predictor because of its significant correlation with CRP.
