# Supplementary figures and images for: The role of microbiota and inflammation in self-judgement and empathy: implications for understanding the brain-gut-microbiome axis in depression
Source: Psychopharmacology (Berl). 2019 Apr 7;236(5):1459–70. doi: 10.1007/s00213-019-05230-2 (PMC6598942; doi:10.1007/s00213-019-05230-2)

**Supplementary Material – Scatterplots for significant zero-order associations**


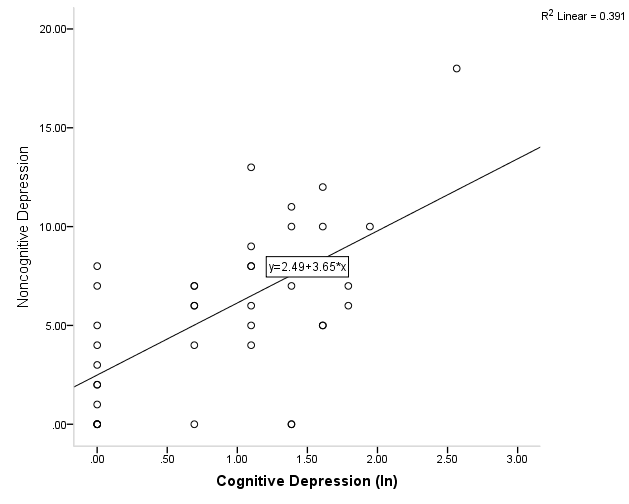

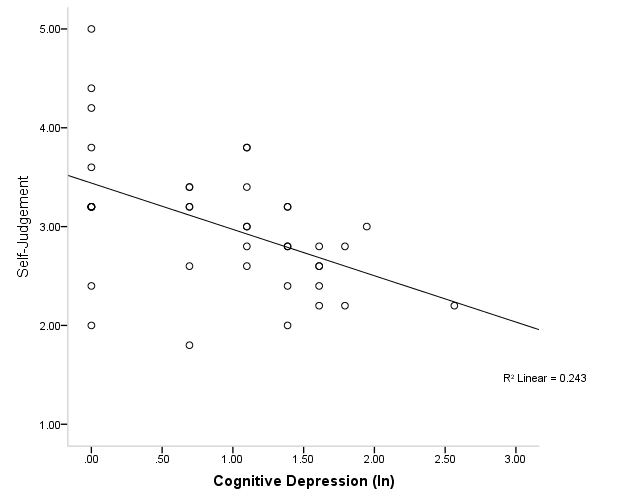


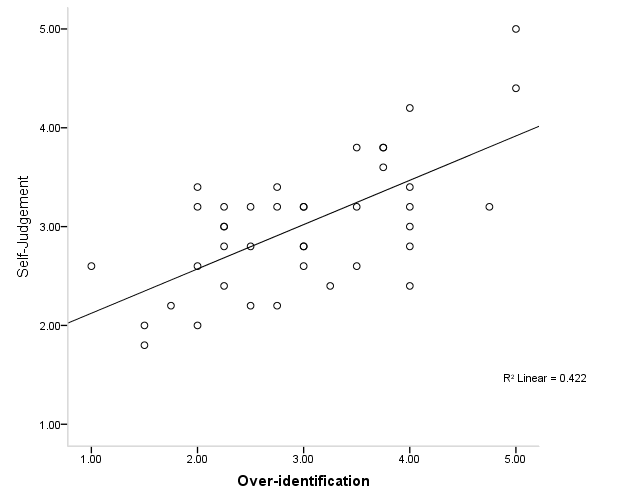

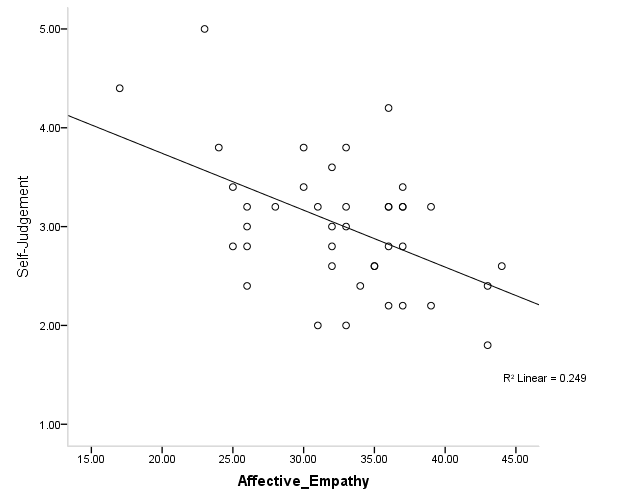


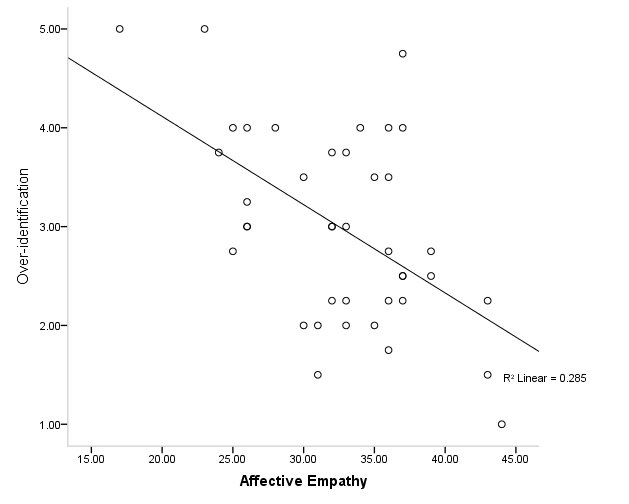

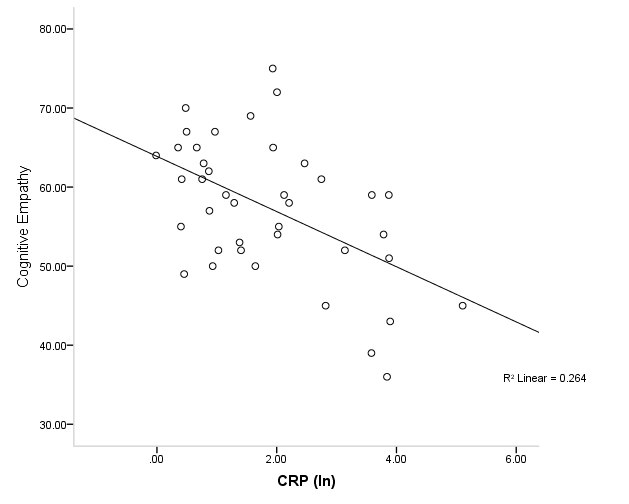


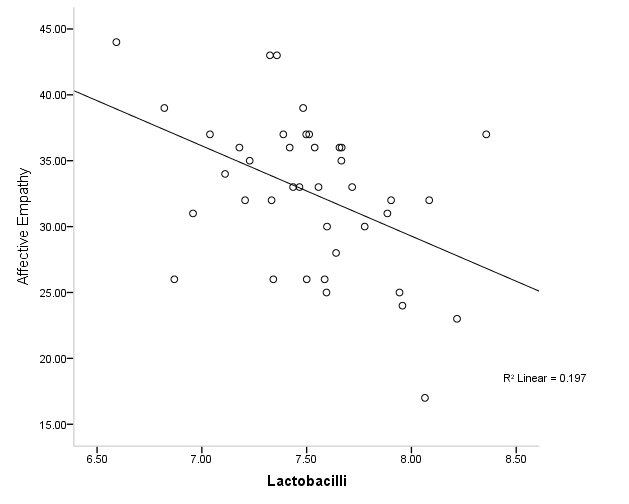

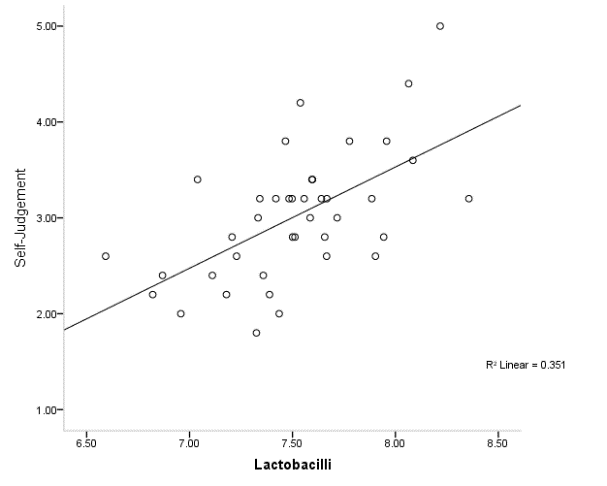

Supplement: Supplementary file 1 — (DOCX 129 kb) [file 213_2019_5230_MOESM1_ESM.docx]
